# Severe Hearing Loss in the Aging Population Poses a Global Public Health Challenge. How Can We Better Realize the Benefits of Cochlear Implantation to Mitigate This Crisis?

**DOI:** 10.3389/fpubh.2019.00227

**Published:** 2019-08-16

**Authors:** Patrick S. C. D'Haese, Vincent Van Rompaey, Marc De Bodt, Paul Van de Heyning

**Affiliations:** ^1^MED-EL GmbH, Innsbruck, Austria; ^2^Antwerp University Hospital – University of Antwerp, Antwerp, Belgium; ^3^Faculty of Medicine and Health Sciences, University of Antwerp, Antwerp, Belgium; ^4^Department of Speech, Language and Hearing Sciences, Ghent University, Ghent, Belgium

**Keywords:** hearing loss, hearing aids, cochlear implants, awareness, policy

## Introduction

An astonishing 466 million people across the globe live with a disabling hearing loss, equating to 6.1% of the world's population (1). As our global population continues to age, with the 65 plus age group expected to grow from 534 million in 2010 to nearly 1.5 billion in 2050, the number of those experiencing hearing loss is expected to increase (2), primarily because its most common cause in adults is the ordinary process of aging. It is predicted that by 2050 more than 900 million people will have disabling hearing loss (1), bringing with it an array of public health challenges including increased and costly co-morbidities such as cognitive decline, type 2 diabetes, more frequent falls and social isolation, which come at a significant economic and social cost. In fact, it is estimated by the World Health Organization (WHO) that unaddressed hearing loss at any age poses an annual global cost of 750 billion dollars (3), highlighting the economic costs of non-treatment. Good hearing contributes to good quality of life, and access to appropriate treatment ensures citizens can transition smoothly into their older years, helping them to live their lives as healthily as possible: remaining active, retaining independence and contributing to the economy. To do this, awareness of appropriate innovative and cost-effective hearing technologies, such as Cochlear Implants (CI) for severe or profound hearing loss, must be raised, recognized as a human-right, and barriers to receiving them must be alleviated.

## Focusing on the Public Health Challenges

To first understand the severity of the challenge of severe hearing loss in the aging population, and understand the necessity of appropriate treatment, it is important to explore the costly co-morbidities that are associated with it.

According to the Lancet Commission, untreated hearing loss is the number one modifiable risk factor contributing to dementia, increasing an individual's risk of developing the condition by 9% (4). This trend is prevalent from mid through to late-life, even with mild levels of hearing loss. One explanation is that untreated hearing loss increases the cognitive resources required by the brain to convert sound into information. This depletes the cognitive reserve available for processes such as working memory (4, 5). Most dementia patients require extensive care and support with even the most basic daily activities, leading to a heavy economic and social burden.

Hearing loss has an impact on an individual's mental health and is also linked to the onset of depression. Social situations become more challenging through inhibited communication, leading to social withdrawal and isolation. This can further contribute to cognitive decline through depriving the brain of the stimulation it gets from social interactions (4, 5). Cognitive decline and social isolation as a result of untreated hearing loss lead to increased hospitalization rates and poorer self-reported health for individuals over the age of 70 (5).

Finally, the treatment and management of diabetes is hindered through hearing loss, impacting diabetes education through hindering communication between doctor and patient, making self-management of the condition more difficult (6).

Considering the aforementioned co-morbidities and the fact that untreated hearing loss is also related to more frequent falls through reduced balance and restricted environment awareness, we can start to see how hearing loss might accelerate the deterioration of an individual's health and their progression into assisted living. Unfortunately, this can deprive people of the joy of their golden years, reduces independence, and often comes at significant financial costs. This makes hearing loss a significant public health challenge.

## The Benefits of Cochlear Implantation and Barriers to Access

Cochlear implantation has become a standard treatment for adults and children with bilateral severe to profound hearing loss, when powerful hearing aids no longer provide enough functional benefit toward speech understanding. Whilst surgically implanted, they can be recommended for even the oldest of adults. Numerous scientific studies have shown that hearing implants positively affect many factors. Older adults experience improved speech understanding, increased social contact, greater self-confidence, and an overall improvement in quality of life after implantation (7). Multiple studies have shown that effective treatment of hearing loss can reduce or even nullify the increased risk of cognitive decline and that individuals who receive treatment over an extended period of time have no increased risk of developing depression. Cochlear implants have been shown to be highly cost-effective, with low complication rates, and in most developed economies, funding is provided by national health programmes, employer-based insurance, or private insurance schemes (8).

However, as of December 2012, only a fraction of those who could benefit from Cochlear Implantation were reported to have received a CI: only 324,200 people world-wide. Across all regions, less than 10% of those with severe to profound hearing loss have been implanted (8), with alarming statistics of ~1% in Japan for adults and children (9), and 0.3% in Australia for the age group 65 to 74 years (10). Conversely, for hearings aids the average statistic for use in the severe to profound range sits at 70 to 90% (8). This has led researchers to explore why the uptake of CI is so low, despite its benefits and lack of awareness has been identified as a key theme that must be addressed.

First, the low uptake of CI is in part due to the low number of suitable candidates presenting themselves for assessment. People have to be motivated enough to seek help for their hearing loss, and typically only do so when they are sufficiently concerned about its severity or that treatment on balance would be more beneficial than detrimental. With the older population specifically, this can be a barrier given that hearing loss can be viewed as a natural consequence of old age, by both the patient and professionals (8). Concerns regarding surgery, loss of residual hearing and rehabilitation can be additional barriers to adults deciding that they would like to be referred for a CI assessment.

There is also a variance between those who are appropriate for CI and those who receive them, depending on the approach to hearing care across different countries. For example, in the United Kingdom, the first point of access for hearing care is the General Practitioner (GP) (83%), however in Germany for example, it is an Ear Nose and Throat (ENT) specialist (93%) (8). GP management of age-related hearing impairment was found to be a barrier to seeking help for hearing impairment.

Furthermore, studies reveal that there is a lack of awareness as to whether Cochlear Implantation is an option for the older population, despite the fact that the surgical complication rate is low even for older adults, if the patient's general state of health is good (11). This is preventing eligible candidates from seeking appropriate and potentially life changing treatment.

Finally, an issue beyond awareness which cannot be excluded is the topic of payment. Global inequalities currently exist between high, low and middle income countries, in terms of how Cochlear Implants are reimbursed and funded. The cost of Cochlear Implants must not be a barrier to receiving treatment, and policy-makers must recognize access to hearing health as a human right.

## Conclusion

To conclude, hearing loss carries a heavy economic and social burden, which is only expected to increase as our global population continues to age. Hearing loss has a proven negative impact on an individual's overall health. If we do not provide access to appropriate treatment costly co-morbidities such as cognitive decline, depression and the mismanagement of diabetes will continue to deprive our older population of experiencing the full potential of their golden years, whilst placing huge financial costs on society. Cochlear Implantation is a cost-effective solution to alleviating the pressure of hearing loss on both individuals and global health systems and is appropriate for even older adults with low surgical complication rates. However, awareness amongst patients and professionals of Cochlear Implantation remains a key barrier to access for potential candidates, with alarmingly low rates of penetration. Hearing loss should not be viewed as a natural part of aging without a solution. To realize the full benefits of Cochlear Implantation, awareness of the CI must be raised amongst patients and professionals as well as policy-makers so that equitable reimbursement takes place across the globe.

## Author Contributions

All authors contributed to the development of the opinion, the interpretation of existing public health policies related to hearing loss and the review of the final paper.

### Conflict of Interest Statement

PD is an employee of MED-EL GmbH, Innsbruck, Austria. PV receives grants to the institution from MED-EL and Cochlear. The remaining authors declare that the research was conducted in the absence of any commercial or financial relationships that could be construed as a potential conflict of interest.
